# Survey of emergency tracheostomy management in the East of England region

**DOI:** 10.1186/cc12105

**Published:** 2013-03-19

**Authors:** N Lawrence, L Oakley, C Swanavelder, M Palmer

**Affiliations:** 1 West Suff olk Hospital, Bury St Edmunds, UK

## Introduction

The 4th National Audit Project of the Royal College of Anaesthetists [1] concluded that the majority of airway-related significant complications in ICUs resulted from displaced or blocked tracheostomies and recommended together with the Intensive Care Society and the National Tracheostomy Safety Project that each ICU in the UK should have an emergency airway management plan and guidelines [2]. The aim of this survey was to establish whether such guidelines exist and are familiar to those working within the ICUs of the East of England (EoE), their ease of availability in an emergency and the degree of emergency tracheostomy training within the region.

## Methods

Data collection was via a telephone survey of 11 ICUs in the EoE training region during July 2012 with one senior ICU nurse and one ICU trainee questioned per hospital. Questions related to the existence and accessibility of guidelines for tracheostomy emergencies, and to the respondent's degree of emergency tracheostomy training and their perceived availability of formal training.

## Results

All 11 ICUs questioned perform and manage tracheostomies. Of 22 respondents, 10 knew of guidelines covering all of the emergencies described above and their location. Four respondents thought that these guidelines were accessible in an emergency setting, one-half of which were on computer systems requiring a login and search function. With regards to emergency management, 19 respondents felt competent in a tracheostomy emergency; almost exclusively through experience and in-house teaching. No respondents were aware of any formal emergency tracheostomy management courses.

## Conclusion

Despite national guidance within the UK this survey highlights that implementation and awareness of emergency tracheostomy guidelines in ICUs in the EoE region is poor, and when present they are not readily accessible in an emergency. Emergency training has largely been informal and the availability of formal training courses has not been recognised. In order to improve patient safety there is a need to ensure that emergency tracheostomy management including guidelines, equipment and formalised tracheostomy emergency training are adopted and embraced universally.
